# Immunological characterization of *Plasmodium vivax* Pv32, a novel predicted GPI-anchored merozoite surface protein

**DOI:** 10.1186/s12936-018-2401-7

**Published:** 2018-07-27

**Authors:** Yang Cheng, Bo Wang, Feng Lu, Jin-Hee Han, Md Atique Ahmed, Eun-Taek Han

**Affiliations:** 1 Department of Public Health and Preventive Medicine; Laboratory of Pathogen Infection and Immunity, Wuxi School of Medicine, Jiangnan University, Wuxi, Jiangsu People’s Republic of China; 2 Department of Medical Environmental Biology and Tropical Medicine, School of Medicine, Kangwon National University, 1 Kangwondaehakgil, Chuncheon, Gangwon-do 24341 Republic of Korea; 3 Department of Clinical Laboratory, The First Affiliated Hospital of Anhui Medical University, Hefei, Anhui People’s Republic of China; 4 Department of Pathogen Biology and Immunology, School of Medicine, Yangzhou University, Yangzhou, People’s Republic of China

**Keywords:** *Plasmodium vivax*, Pv32, Predicted GPI-anchored protein, Merozoite surface protein, Immune response

## Abstract

**Background:**

The development of an effective malarial vaccine is an urgent need. Most glycosylphosphatidylinositol (GPI)-anchored proteins of *Plasmodium* parasites are exposed to neutralizing antibodies, and several are advanced vaccine candidates. In the present study, *Plasmodium vivax* Pv32 (PVX_084815) as a hypothetical, predicted GPI-anchored and cysteine-rich motif was identified from our previous findings with a focus on its antigenic profiling. The orthologue gene *pv32*, a predicted GPI anchor of *P. falciparum* PF3D7_1434400, has still not been well studied.

**Methods:**

The gene information of *pv32* was obtained from PlasmoDB. Recombinant Pv32 protein was expressed and purified using a wheat germ cell-free expression system and a glutathione-Sepharose column. Naturally acquired immune response to recombinant Pv32 protein was evaluated using a protein microarray with 96 parasite-infected patients and 96 healthy individuals. Antibodies against recombinant Pv32 proteins from immune animals were produced, used and analyzed for the subcellular localization of native Pv32 protein by an immunofluorescence assay. A total of 48 *pv32* sequences from 11 countries retrieved from PlasmoDB were used to determine the genetic diversity, polymorphisms and genealogical relationships with DNAsp and NETWORK software packages.

**Results:**

*Pv32* is encoded by a conserved gene with two introns that are located on chromosome 13 and expressed as a 32 kDa protein in mature asexual stage parasites. Immunofluorescence data showed that Pv32 localized on the merozoite surface in schizont-stage parasites. The recombinant Pv32 was recognized by 39.6% of antibodies from *P. vivax*-infected individuals compared with healthy individuals. Low levels of nucleotide diversity (π = 0.0028) and polymorphisms of *pv32* were detected within worldwide isolates.

**Conclusions:**

This study shows the identification and characterization of the hypothetical protein, Pv32. Pv32 provides important characteristics, including a merozoite surface protein, a predicted GPI motif and Cysteine-rich motif among *Plasmodium* species. These results suggested that Pv32 is immunogenic with a merozoite surface pattern to antibodies during natural infection in humans.

**Electronic supplementary material:**

The online version of this article (10.1186/s12936-018-2401-7) contains supplementary material, which is available to authorized users.

## Background

Although infection by *Plasmodium vivax* has been called “benign tertian malaria”, it poses as a major threat to health in South and Southeast Asia, as well as in South America, where 2.6 billion people are at risk, resulting in more than a hundred million malarial infections annually [1, 2]. As a neglected parasite, only little is known regarding the pathobiology of *P. vivax* malaria [3]. This understanding may be limited by the following: (1) the difficulty of long-term culturing of *P. vivax* may not be applicable for their functional analysis; (2) the standardized human/or animal challenge model was not well developed for pre-clinical trials of vivax vaccine candidates; and (3) *P. vivax* genomic diversity limits the discovery of a *P. vivax* vaccine roadmap.

In *Plasmodium* parasite pathobiology, invading and modifying human erythrocytes are essential processes [4]. Most of the proteins that play key roles in invasion are either stored in the apical secretory organelles or located on the surface of the merozoite. On the parasite membrane surface, some merozoite surface proteins anchor into the merozoite surface via the glycosylphosphatidylinositol (GPI)-tail and form complexes with other non-covalently associated proteins, such as MSP6, MSP7 and Pf41 [5–7]. In addition, merozoite surface proteins are a direct factor in inducing host immune response during parasitic invasion into erythrocytes after being ruptured. In parasite-infected patients, antibodies to these proteins probably confer host protection by inhibiting parasite invasion, blocking intraerythrocytic parasites, and inducing mononuclear cell-mediated inhibition [8].

Among our previous findings, a predicted GPI-anchored protein, Pv32, has been identified for its reactions with *P. vivax* exposed sera [9]. The homologue of Pv32, *P. falciparum* hypothetical protein (Pf32; PF3D7_1434400 and PF14_0325), has been expressed, and anti-Pf32 antibody reacted with blood-stage parasites [10]. Although it was not clearly defined, its subcellular localization and functional activity as a Cysteine-rich protein may play a role during parasite invasion. Pv32 still needs to be further characterized for understanding the *P. vivax* malarial pathobiology. In this study, a predicted-GPI anchor Pv32 recombinant protein was successfully expressed and purified by a wheat germ cell-free (WGCF) expression system on the basis of the *P. vivax* Sal-I strain sequence. Antibody response was evaluated from clinical vivax-infected patients compared to healthy individual sera. Subcellular localization of native Pv32 was determined in the blood-stage parasites with immune serum by an immunofluorescence assay. The genetic diversity, polymorphisms and genealogical relationships of *pv32* were also analyzed from worldwide isolates.

## Methods

### Gene identification and protein sequence analysis

*Pv32* sequence data and gene expression profiles were analyzed from previous reports [11, 12] using the PlasmoDB website (http://plasmoDB.org; Accession No. PVX_084815). Predicted protein domains were further analyzed using the Simple Modular Architecture Research Tool (SMART) (http://smart.embl-heidelberg.de/) and SOSUIsignal (http://bp.nuap.nagoya-u.ac.jp/sosui/). Amino acid sequence identity between Pv32 and its orthologous sequences in *P. falciparum* (PF3D7_1434400)*, P. ovale curtisi* (PocGH01_13025900)*, P. malariae* (PmUG01_13025900) and *P. knowlesi* (PKNH_0421000) were determined using the Clustal W alignment program in Lasergene 7.0 (Dnastar Inc., Madison, WI, USA). Phylogenetic analysis was conducted using the maximum likelihood method on the Poisson correction model with 1000 bootstraps in MEGA v5.0 software.

### Human serum samples

Positive serum samples for *P. vivax* malaria were collected from 96 patients (mean age, 34 years; range 3–79 years), who had symptoms including fever and parasitemia, by Giemsa-stained thin blood smear microscopy in 50 fields (mean parasitemia, 0.149%; range 0.008–0.750%) for *P. vivax* malaria at local health centers and clinics in endemic areas of the Republic of Korea (ROK). Ninety-six sera samples from healthy, microscopically negative individuals were collected from non-endemic areas of ROK and used for immune response analysis. This study was approved by the Institutional Review Board at Kangwon National University Hospital (IRB No. 2014-08-008-002).

### Expression and purification of recombinant Pv32 (rPv32)

*Pv32* was designed from the *P. vivax* Sal-I strain sequence (PlasmoDB, PVX_084815) and was amplified from the genomic DNA of *P. vivax* isolates from ROK. Genomic DNA was prepared as described previously [9]. Gene coding of *pv32* was amplified using genomic DNA with in-fusion cloning primers, Pv32_F (3′-GGGCGGATAT*CTCGAG*GCAGGAGGCGTTTCCGA-5′) and Pv32_R (3′-GCGGTACCCG*GGATCC*TCAATTCTTGGGGTTACAAAACAAGTC-5′). The vector sequences are underlined, and the restriction enzyme sites (*Xho*I for the sense primer and *BamH*I for the anti-sense primer) are in italics. We expressed and purified rPv32, which lacked the signal peptide and GPI motif, by using WGCF expression [9]. Briefly, amplified DNA fragments were cloned into the pEU-E01-GST-TEV-MCS-N2 vector (CellFree Sciences, Matsuyama, Japan), and the cloned inserts were sequenced using an ABI 3700 Genetic Analyzer (Genotech, Daejeon, Korea) as previously described [13]. The glutathione *S*-transferase (GST) fusion protein was expressed using a WGCF system and was purified with a glutathione-Sepharose 4B column, according to the manufacturer’s instructions (GE Healthcare, Camarillo, CA, USA). Recombinant GST protein was purchased from Abcam (Cambridge, UK) and used for western blot and protein array as a control for GST-fusion protein.

### Animal immunization with recombinant *P. vivax* merozoite surface protein 1-19 (rPvMSP1-19) and rPv32

Female BALB/c mice (DBL Co., Seoul, ROK) were used at 6–8 weeks of age. Three mice were injected intraperitoneally with approximately 30 µg of rPvMSP1-19 and phosphate buffered saline (PBS) with Freund’s complete adjuvant (Sigma-Aldrich, St. Louis, MO, USA). Booster injections were given 3 and 6 weeks later using the same amount of antigen with Freund’s incomplete adjuvant (Sigma-Aldrich). Mouse blood samples were taken 2 weeks after the last booster.

To generate antibodies against Pv32 for the immunofluorescence assay, one Japanese white rabbit was immunized subcutaneously with 250 μg of purified proteins and Freund’s complete adjuvant, as well as with 250 μg with Freund’s incomplete adjuvant thereafter. Immunizations were done 3 times at 3-week intervals. The antiserum was collected 14 days after the last immunization. All animal experimental protocols were approved by the Institutional Animal Care and Use Committee of Kangwon National University, and the experiments were conducted according to the Ethical Guidelines for Animal Experiments of Kangwon National University (KIACUC-16-0158).

### SDS-PAGE and western blot analysis of rPv32

rPv32 was separated using 12% SDS-PAGE after denaturation with the reducing agent β-mercaptoethanol in sample buffer and then stained with Coomassie brilliant blue. For western blot analysis, recombinant proteins were transferred electrophoretically to PVDF membranes (Millipore Corp., Bedford, MA, USA) and incubated with blocking buffer (5% non-fat milk in PBS containing 0.2% Tween 20 and PBS/T) for 1 h at 37 °C. After blocking, either anti-GST antibody, mouse and rabbit immune sera or mixed patient sera was diluted with PBS/T 200 times. Secondary IRDye^®^ goat anti-mouse (1:10,000 dilution), IRDye^®^ goat anti-rabbit (1:20,000 dilution) or IRDye^®^ goat anti-human (1:20,000) (LI-COR^®^ Bioscience, Lincoln, NE, USA) were used to detect GST-tagged recombinant protein and immune serum of a specific quality. Sera from healthy people from the ROK and PBS-immunized rabbit serum were used as controls. Data were scanned with an Odyssey infrared imaging system (LI-COR Biosciences, Lincoln, NE, USA) and analyzed by Odyssey software (LI-COR, Inc.).

### Indirect immunofluorescence assay (IFA)

The schizont stage-rich parasites obtained from short-term in vitro culture were spotted onto multi-well slides and fixed with ice-cold acetone for 3 min, dried, and stored at − 80 °C. Before use, the slides were thawed on silica gel blue (Samchun Chemical, Pyeongtaek, Gyeonggi, ROK) and blocked with PBS containing 5% non-fat milk at 37 °C for 30 min. The slides were incubated with 1:200 diluted primary antibodies (mouse anti-PvMSP1-19 and rabbit anti-Pv32) at 37 °C for 1 h. The PvMSP1-19 was used as the merozoite surface protein marker. After the primary antibody reactions, the slides were stained with Alexa 546-conjugated goat anti-rabbit IgG secondary antibody (Ab) or Alexa 488-conjugated goat anti-mouse IgG secondary Ab (Invitrogen Corp., Carlsbad, CA, USA), and nuclei were stained with 4′,6-diamidino-2-phenylindole (DAPI, Invitrogen Corp.) at 37 °C for 30 min. The slides were mounted with ProLong Gold antifade reagent (Invitrogen Corp.) and viewed under oil immersion with a confocal laser scanning FV200 microscope (Olympus, Tokyo, Japan) equipped with 20× dry and 60× oil objectives. Images were captured with FV10-ASW 3.0 viewer software and prepared for publication with Adobe Photoshop CS5 (Adobe Systems, San Jose, CA, USA).

### Protein arrays

Amine coated slides and protein arrays were prepared as described in previous studies [14–16]. Briefly, serum samples from 96 people with *P. vivax* malaria and 96 unexposed individuals were used for humoral immune response analysis. Purified rPv32 was spotted into duplicate wells for arrays at 25 ng/μl in PBS and incubated for 1 h at 37 °C. After blocking with 1.0 μl of blocking buffer (5% BSA in PBS with 0.1% Tween 20, PBS/T) for 1 h at 37 °C, the chips were probed with human sera from malaria patients or healthy individuals (1:50 dilution) that were first pre-absorbed against wheat germ lysate (1:100 dilution) to block anti-wheat germ antibodies. Samples were detected by Alexa Fluor 546 goat anti-human IgG (10 ng/μl, Invitrogen Corp.) in PBS/T, quantified as described previously and scanned by a fluorescence scanner (ScanArray Express, PerkinElmer, Boston, MA, USA) [14]. The cut-off value is equal to the mean ± three standard deviations (SD) of the mean fluorescence intensity (MFI) of the 96 negative samples.

### Sequence diversity, polymorphisms, and haplotype analysis of *pv32* from worldwide isolates

Forty-eight *pv32* sequences (718 bp, excluding the signal peptide and GPI motif) from 11 countries (Mexico, Peru, Thailand, Papua New Guinea, Brazil, Colombia, India, North Korea, China, Madagascar and Bolivia) along with the Sal-1 strain were retrieved from the PlasmoDB database (Additional file 1: Table S1). Sequences were aligned using the CLUSTAL-W program in MegAlign Lasergene ver. 7.0 (DNASTAR). Sequence diversity (π) was defined as the average number of nucleotide differences per site between two sequences, and the number of polymorphic sites was determined using the DNAsp ver. 5.0 software. The relationships among the haplotypes of *pv32* were evaluated with the median-joining method using the NETWORK software ver. 4.6.1.2. (Fluxus Technology Ltd., Suffolk, UK).

### Statistical analysis

The data were analyzed using GraphPad Prism (GraphPad Software, San Diego, CA) and SigmaPlot (Systat Software Inc., San Jose, CA, USA). Mann–Whitney *t*-tests were used to compare the differences between the means of each group for statistical significance. Statistical differences of *p* < 0.05 were considered significant. Simple scatter-regression was used to make the standard curve.

## Results

### Expression of rPv32 and reactivity to immune sera

**Fig. 1:**
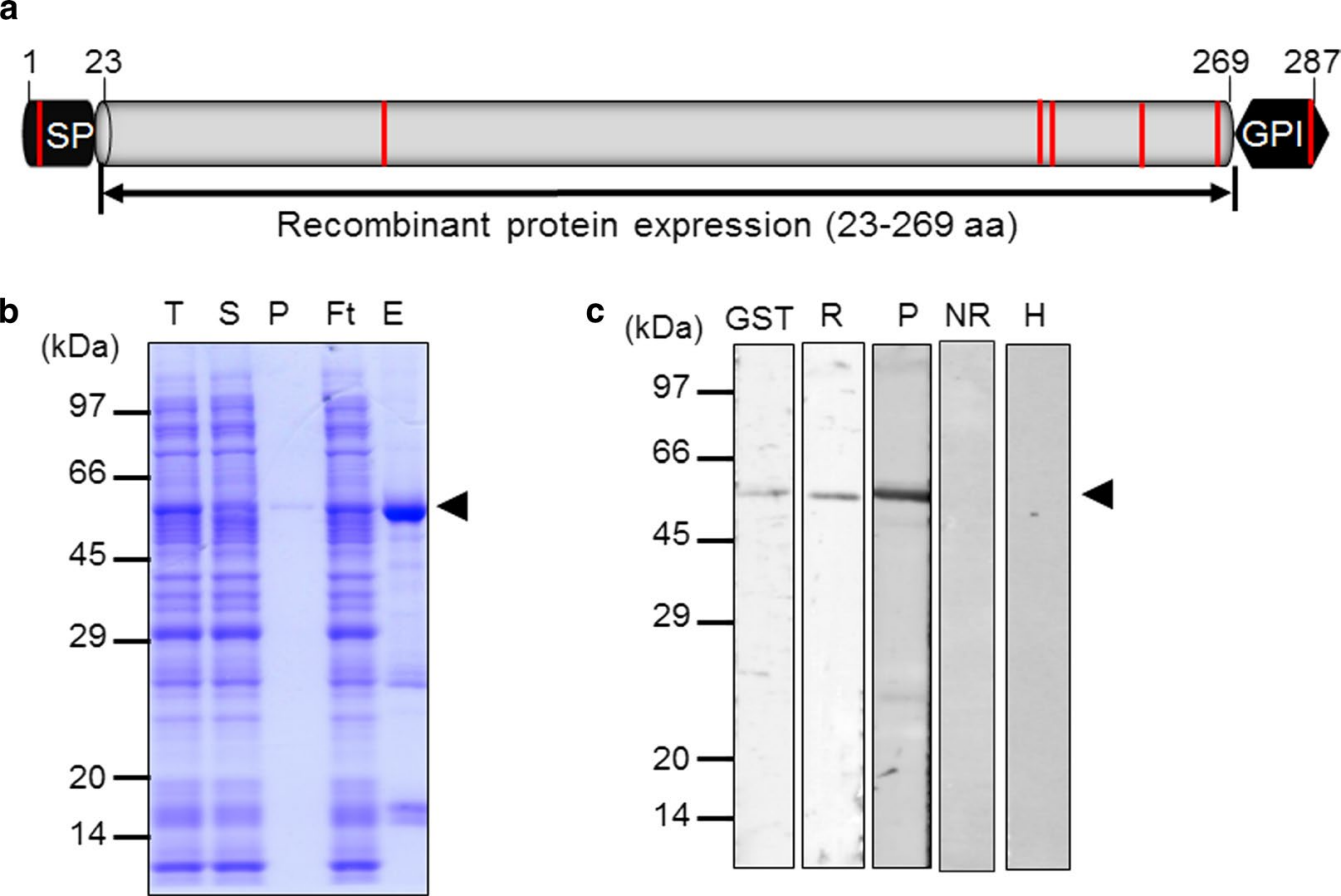
Schematic diagram of Pv32, recombinant Pv32 protein expression and immunization. **a** Schematic diagram of Pv32. The Pv32 protein comprises 287 amino acids, with a calculated molecular mass of 32 kDa. The signal peptide (amino acid [aa] position 1–22) and GPI motif (aa, 270–287) are indicated. Truncated Pv32 (23–269 aa) was constructed for expression. The red bar indicates the cysteine residue position. *SP* signal peptide, *GPI* glycosylphosphatidylinositol. **b** The purification progress of Pv32 (58 kDa) was resolved by 12% SDS-PAGE. *T* total, *S* supernatant, *P* pellet, *Ft* flow-through, *E* elution. **c** Western blot analysis of recombinant Pv32 with anti-GST antibody (GST), rabbit immune sera (R), pooled *P. vivax* patient sera (P), PBS-immunized rabbit sera (NR), and non-infected human sera (H). Arrowheads indicate specific bands for each recombinant protein

Pv32 consists of 287 residues in the Sal-I strain, including a hydrophobic SP within its first 22 amino acids, and a predicted GPI motif within the last 18 amino acids (Fig. 1a). Pv32 has 7 cysteine residues, including one in the SP and one in the predicted GPI motif. Pv32 has amino acid sequence identities of 80.8, 61.6, 54.7, and 52.8% with *P. knowlesi* (PKNH_0421000), *P. ovale* (PocGG01), *P. falciparum* (PF3D7_1431100) and *P. malariae* (PmUG01_13025900), respectively. Thus, in the phylogenetic analysis, Pv32 was in the same clade as *P. knowlesi*. Moreover, *P. malariae* and *P. ovale curtisi* also were in the same clade, but Pv32 was located relatively far from *P. falciparum* (Additional file 2: Figure S1).

The purity and protein folding of rPv32 without SP and the predicted GPI motif expressed by the WGCF expression system were assessed (Fig. 1a). The truncated rPv32 (∆SP/∆GPI) was purified under non-denaturing conditions as shown in Fig. 1b. The integrity and purity of the purified recombinant proteins was assessed by SDS-PAGE. Purified rPv32 migrated as a single band of ~ 58 kDa in reducing conditions corresponding to 26 kDa tagged GST and expected rPv32 molecular weight (32 kDa). The corresponding immunoblots were probed with an anti-GST tag monoclonal antibody (GST), anti-Pv32 rabbit immune serum (R), and pooled *P. vivax* patient sera (P). The PBS-immune rabbit sera (NR) and the malaria-naïve human serum samples (H) obtained from individuals living in malaria-free regions of Gangwon, ROK were used as negative controls, and there was no reactivity in this group (Fig. 1c).

### Analysis of humoral immune response to Pv32

To evaluate the humoral immune response against purified rPv32, the antibodies present in human sera were screened with purified rPv32 protein by protein array analysis. Antibody responses against Pv32 in serum samples from 96 patients infected with *P. vivax* and 96 healthy individuals were determined. The sera from *P. vivax* exposed individuals showed a significantly higher MFI of total IgG than did sera from non-infected healthy subjects. The prevalence of anti-Pv32 antibodies showed a sensitivity of 39.6% (38 in 96 vivax samples > cut-off value) and a specificity of 99.0% (9 in 96 healthy samples < cut-off value) compared with GST only (Table 1). These data confirmed the potential and considerable immunogenicity of Pv32, which may be exposed to the host immune system by surface protein localization.

**Table 1:** IgG responses to recombinant Pv32 and GST control proteins in the sera of vivax patients and healthy individuals

Protein	No. of patient samples (*n*)			MFI^e^	95% CI^b^	No. of healthy samples (*n*)			MFI^e^	95% CI^b^	*p* value^d^
	Positive	Negative	Total (%)^a^			Positive	Negative	Total (%)^c^			
Pv32	38	59	96 (39.6)	6252	29.8–49.4	1	95	96 (99.0)	2879	97.0–100	< 0.0001
GST	0	30	30 (0.0)	2895		0	30	30 (100)	2880		*ns*

^a^Sero positive rate: % of positive in-patient samples

^b^Confidence intervals

^c^Sero negative rate: % of negative in healthy samples

^d^Differences in the total IgG prevalence for each antigen between vivax patients and healthy individuals were calculated with Student’s t-test, *p *< 0.05 considered as statistically significant; ns, not significantly different

^e^MFI: mean fluorescence intensities were divided by cut off value + standard deviation above the mean fluorescence intensity of the malaria naïve samples

### Pv32 localizes on the merozoite surface

**Fig. 2:**
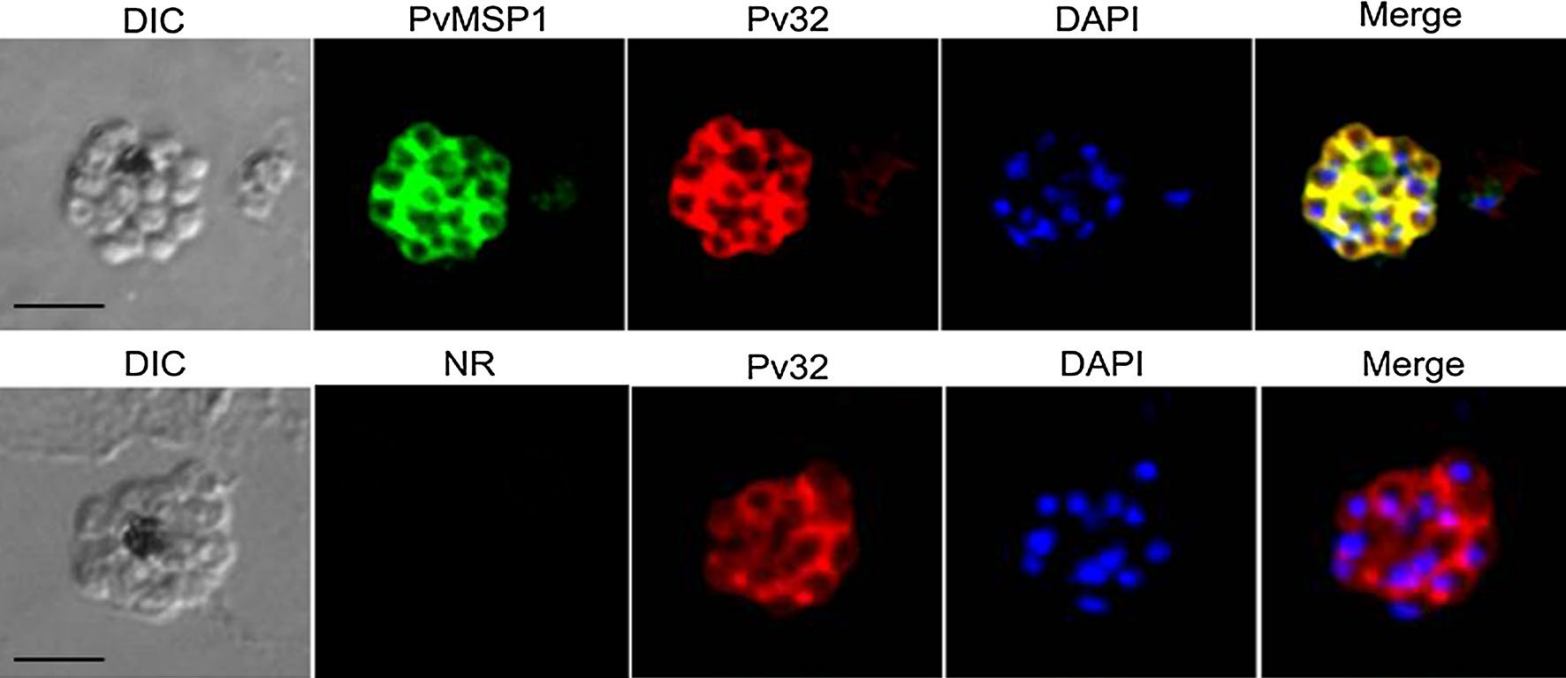
Localization of Pv32 in mature schizont-stage parasites. Schizont-stage parasites were dual-labeled with rabbit antisera against Pv32 (red) and PvMSP1-19 (merozoite surface marker, green). Nuclei are stained with DAPI in the merged images. Scale bars represent 2.5 µm. DIC, differential interference contrast

To know the localization of Pv32 in native parasites, the anti-PvMSP1 antibody was used as protein surface marker by IFA. Pv32 was highly transcribed and expressed in the schizont stage throughout the 48-h intraerythrocytic cycle according to the PlasmoDB database; therefore, in this study, we found that Pv32 completely overlapped with PvMSP1 surface marker (Fig. 2). Hence, it suggested that Pv32 is a merozoite surface protein.

### Sequence analysis of *pv32* from worldwide isolates

**Fig. 3:**
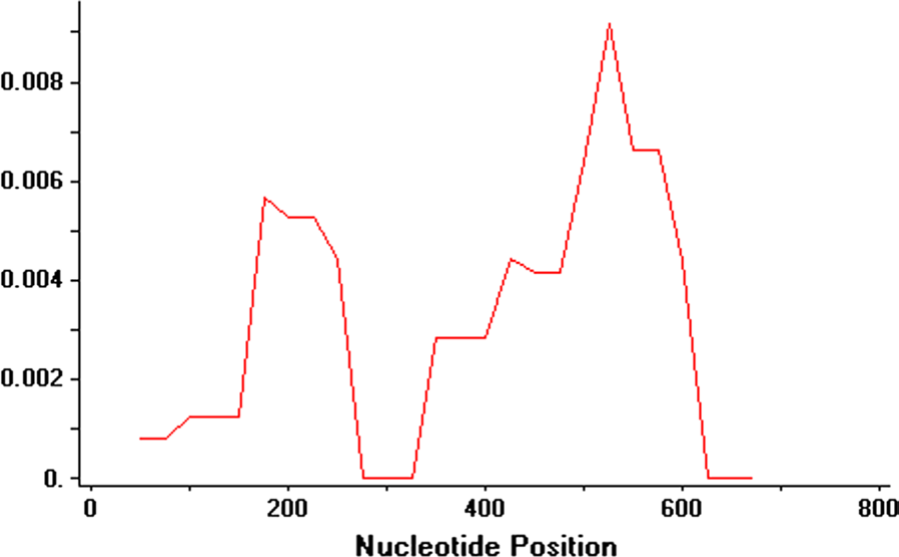
Sliding window (size = 100 bp, length = 25 bp) analysis of genetic diversity (π) across the *pv32* gene from worldwide *P. vivax* isolates

**Fig. 4:**
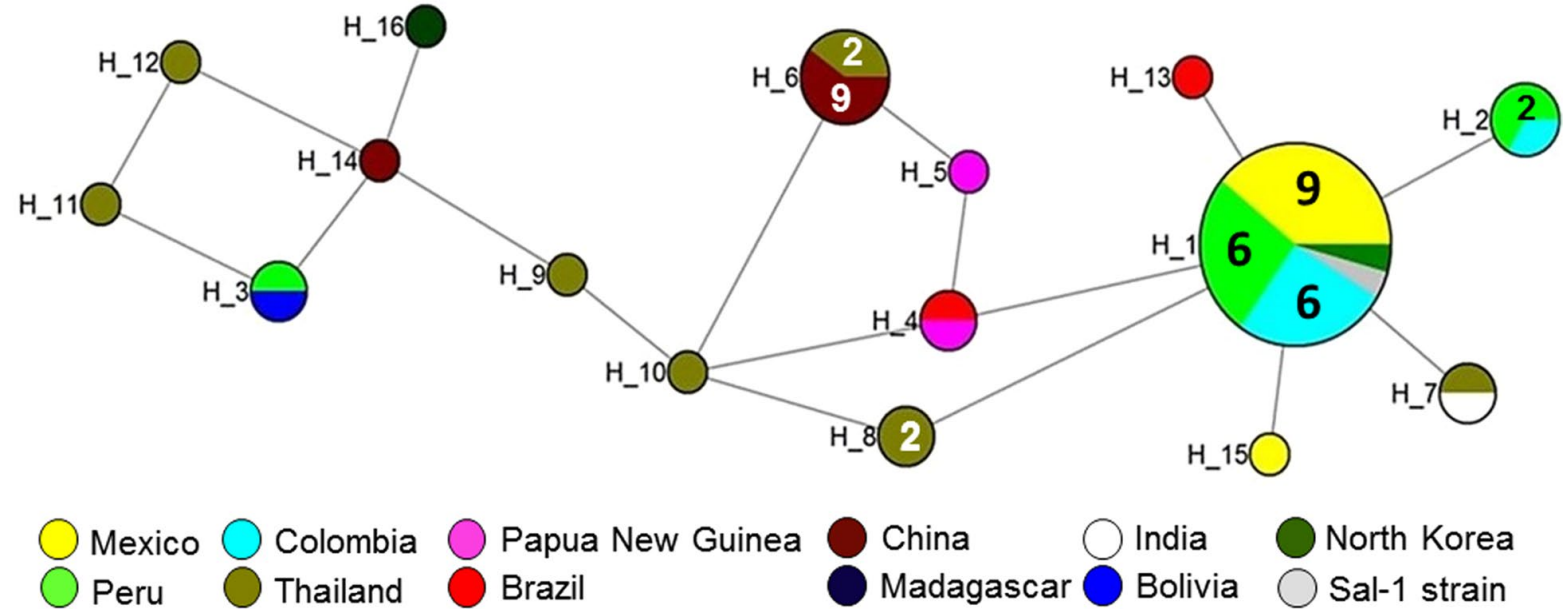
Median-joining networks of 16 *pv32* haplotypes of *P. vivax* isolates from 11 countries. The genealogical haplotype network showing relationships among 16 *pv32* haplotypes of *P. vivax* from 11 countries. ‘H’ numbers designate the haplotype number, the size of each circle represents the frequencies of the haplotype, and unnumbered circles represent a single haplotype. Geographical haplotypes are indicated by the color key. Distances between nodes are arbitrary

The 48 *pv32* sequences exhibited a low level of diversity (π = 0.00280) comparable to published *P. vivax* merozoite surface proteins [17, 18] (Fig. 3; Table 2). Alignment of the *pv32* sequences revealed 11 (1.53%) polymorphic and 707 (98.46%) invariant sites. The haplotype network analysis of 48 *pv32* sequences from 11 countries identified 16 unique haplotypes with moderate haplotype diversity (Hd = 0.761) (Table 2; Additional file 3: Table S2). One haplotype (Hap_1) was shared (Fig. 4) by 23 isolates which originated from Mexico (n = 9), Peru (n = 6), Colombia (n = 6) and one each from North Korea and the Sal-1 strain, respectively. Haplotype 6 was shared between Thailand (n = 2) and China (n = 9). Additionally, Haplotype 2, 3, 4, and 7 were at least shared between two countries (Fig. 4).

**Table 2:** Estimates of DNA sequence polymorphism of the *pv32* gene

No. sample (n)	No. polymorphisms	No. haplotypes (h)	Haplotype diversity (Hd)	Nucleotide diversity (π)
48	11	16	0.761	0.00280

## Discussion

Pv32 is a highly conserved cysteine-rich protein found in different *Plasmodium* spp. Pv32 is recognized by sera from individuals naturally infected with *P. vivax*, thus confirming its potential as a vaccine candidate. Its localization and expression during the schizont stage suggest that it has a similar role in host cell invasion to those of other GPI-anchored proteins.

GPI-anchored proteins have been found on the surfaces of extracellular merozoites or apical organelle membranes [7]. Most GPI-anchored merozoite proteins are refractory to genetic deletion, suggesting that they play important roles in blood-stage development [19]. Thirty predicted GPI-anchored proteins have been identified in *P. vivax* [11]. The well-characterized GPI-anchored protein PvMSP1 has been selected as a malaria vaccine candidate for its immunogenic properties in a large proportion of individuals exposed to malaria [20, 21]. The PvMSP1 paralog is a GPI-anchored erythrocyte binding ligand [16] that induces a specific cellular immune response conferring protection against *P. vivax* [22]. Recently, a GPI-anchored micronemal antigen, PvGAMA, has been shown to bind human erythrocytes independently of their Duffy antigen status [23]. Furthermore, the GPI motif of these antigens is thought to be an important factor in inducing proinflammatory responses [24]. In this study, we measured the response frequency to rPv32 in 96 patients with a *P. vivax* mono-infection from an endemic area in the ROK and found that nearly 42.7% of this population had antibodies against Pv32 (Table 1). These data reconfirmed a large number of serum samples as reliable data from previous preliminary findings of Pv32 antigenicity [9]. However, two false positives have been detected in 96 non-exposed samples that may have cross-reacted with some other proteins from healthy individuals. Antibodies are essential for acquired human immunity to malaria. Antibodies are associated with patient age, exposure, active infection and antigens. The immunogenic activity of Pv32 may be because of a parasite surface protein that was frequently exposed to the host immune system. In this study, antibody titers against Pv32 showed median levels of antibody titers, not higher titers compared to other GPI-anchored antigens, such as PvMSP1, PvMSP1P, and PvRAMA from Korean patients [9, 14, 19]. Second, the low endemicity of vivax malaria in ROK also may be related with the low antibody responses from the low frequency of exposure to infective bites in field sites. Thus, functional analysis of Pv32 remains to be investigated regarding whether the anti-Pv32 antibody could inhibit parasite invasion or even be a protective antibody in human patients. Accordingly, *P. knowlesi* could be alternatively used for functional study due to the difficulty of *P. vivax* culture.

This study first described the identification and characterization of a GPI-anchored and cysteine-rich Pv32 as a merozoite surface protein. The characteristics of Pv32 were identified from the conserved gene sequence, the protein’s expression toward the schizont stage and its localization and the broad recognition presented by the sera from individuals infected with *P. vivax.* These data suggest that Pv32 could be a good potential vaccine candidate. Further immunogenicity and protection-inducing ability studies are thus needed in the *Aotus* experimental model to confirm the potential of Pv32-based vaccine against *P. vivax* malaria. As one of the few candidates with minimal polymorphism, it may potentially provide sustained protection against this antigenic variant.

## Additional files

**Additional file 1: Table S1.** Origin of pv32 gene sequences from worldwide isolates.

**Additional file 2: Figure S1.** Molecular phylogenetic analysis by the maximum likelihood method.

**Additional file 3: Table S2.** List of Pv32 haplotypes identified from 11 countries.
