# Supplementary material for: Immunological characterization of Plasmodium vivax Pv32, a novel predicted GPI-anchored merozoite surface protein
Source: Malar J. 2018 Jul 27;17:273. doi: 10.1186/s12936-018-2401-7 (PMC6062930; doi:10.1186/s12936-018-2401-7)
Supplement: Supplementary file 1 — Additional file 1: Table S1. Origin of pv32 gene sequences from worldwide isolates. [file 12936_2018_2401_MOESM1_ESM.pdf]

Table S1. Origin of *pv32* gene sequences from worldwide isolates

Country	Sequence ID
Brazil	Brazil22
	Brazil 32
	Brazil
Bolivia	Bolivia
Colombia	Colombia_30102100437
	Colombia_30102100448
	Colombia_30102100490
	Colombia1
	Columbia_30101099036
	Columbia_30102100485
India	IndiaNYC
	IndiaVII
Madagascar	M08
Mexico	Mexico21A
	Mexico566A
	Mexico760A
	Mexico32-E-03
	Mexico55-03
	Mexico118-A
	Mexico165-A
	Mexico1
	Mexico6308
North korea	Northkorean
Peru	Peru262
	Peru852
	Peru692
	Peru3136
	Peru2025
	Peru07
	Peru260
	Peru3133
	PeruDTS0830
Papua New Guinea	PNG58
	PNG72
Thailand	Thailand
	Thailand_VKBT-72
	Thailand_VKBT-94
	Thailand_VKBT-95
	Thailand_VKBT-100
	Thailand_VKBT-106
	Thailand_VKBT-36
	Thailand_VKBT-39
	Thailand_VKBT-45
China	China_NB_-15
	China_NB-16
	China_NB-17
	China_LZCH-20
