# Supplementary material for: Immunological characterization of Plasmodium vivax Pv32, a novel predicted GPI-anchored merozoite surface protein
Source: Malar J. 2018 Jul 27;17:273. doi: 10.1186/s12936-018-2401-7 (PMC6062930; doi:10.1186/s12936-018-2401-7)
Supplement: Supplementary file 2 — Additional file 2: Figure S1. Molecular phylogenetic analysis by the maximum likelihood method. [file 12936_2018_2401_MOESM2_ESM.pdf]

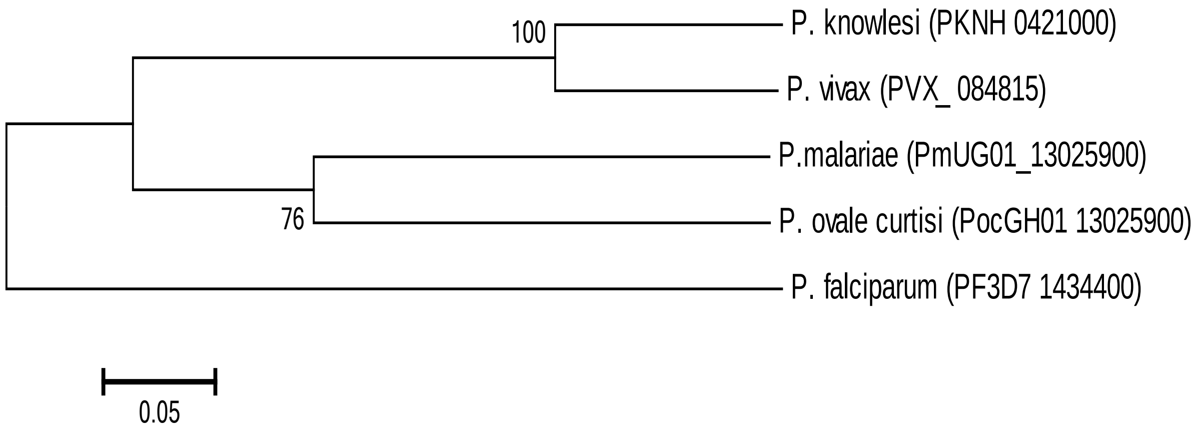

Figure S1. Molecular phylogenetic analysis by the maximum likelihood method. The evolutionary history was inferred by using the maximum likelihood method based on the Poisson correction model [1]. The tree is drawn to scale, and branch lengths are measured by the number of substitutions per site. The analysis involved 5 amino acid sequences. All positions containing gaps and missing data were eliminated. There were a total of 272 positions in the final dataset. Evolutionary analyses were conducted with MEGA 5 software [2].

1. Zuckerkandl E. and Pauling L. (1965). Evolutionary divergence and convergence in proteins. Edited in *Evolving Genes and Proteins* by V. Bryson and H.J. Vogel, pp. 97-166. Academic Press, New York.

2. Tamura K., Peterson D., Peterson N., Stecher G., Nei M., and Kumar S. (2011). MEGA5: Molecular Evolutionary Genetics Analysis using Maximum Likelihood, Evolutionary Distance, and Maximum Parsimony Methods. *Molecular Biology and Evolution* 28: 2731-2739.
