# Supplementary material for: Immunological characterization of Plasmodium vivax Pv32, a novel predicted GPI-anchored merozoite surface protein
Source: Malar J. 2018 Jul 27;17:273. doi: 10.1186/s12936-018-2401-7 (PMC6062930; doi:10.1186/s12936-018-2401-7)
Supplement: Supplementary file 3 — Additional file 3: Table S2. List of Pv32 haplotypes identified from 11 countries. [file 12936_2018_2401_MOESM3_ESM.pdf]

Table S2. List of *Pv32* haplotypes identified from 11 countries

Haplotype no.	Haplotype sequence
Hap_1	CCCGACGCGGG
Hap_2	CCCGACACGGG
Hap_3	CCCGACGCAAA
Hap_4	CCCCACGCGGG
Hap_5	CCCCATGCGGG
Hap_6	CCCCATGCGGA
Hap_7	CCGGACGCGGG
Hap_8	CCCGACGCGGA
Hap_9	CCCCACGCAGA
Hap_10	CCCCACGCGGA
Hap_11	CCCGATGCAAA
Hap_12	CCCCATGCAAA
Hap_13	CTCGACGCGGG
Hap_14	CCCCACGCAAA
Hap_15	GCCGACGAGGG
Hap_16	GCCCCCGCAAA
